# Ultrasensitive and low-volume point-of-care diagnostics on flexible strips – a study with cardiac troponin biomarkers

**DOI:** 10.1038/srep33423

**Published:** 2016-09-16

**Authors:** Nandhinee Radha Shanmugam, Sriram Muthukumar, Shalini Prasad

**Affiliations:** 1 Department of Bioengineering, University of Texas at Dallas, Richardson, TX 75080, USA; 2 EnLiSense LLC, 1813 Audubon Pond Way, Allen, TX 75013, USA.

## Abstract

We demonstrate a flexible, mechanically stable, and disposable electrochemical sensor platform for monitoring cardiac troponins through the detection and quantification of cardiac Troponin-T (cTnT). We designed and fabricated nanostructured zinc oxide (ZnO) sensing electrodes on flexible porous polyimide substrates. We demonstrate ultrasensitive detection is capable at very low sample volumes due to the confinement phenomenon of target species within the ZnO nanostructures leading to enhancement of biomolecular binding on the sensor electrode surface. The performance of the ZnO nanostructured sensor electrode was evaluated against gold and nanotextured ZnO electrodes. The electrochemical sensor functions on affinity based immunoassay principles whereby monoclonal antibodies for cTnT were immobilized on the sensor electrodes using thiol based chemistry. Detection of cTnT in phosphate buffered saline (PBS) and human serum (HS) buffers was achieved at low sample volumes of 20 μL using non-faradaic electrochemical impedance spectroscopy (EIS). Limit of detection (LOD) of 1E-4 ng/mL (i.e. 1 pg/mL) at 7% CV (coefficient of variation) for cTnT in HS was demonstrated on nanostructured ZnO electrodes. The mechanical integrity of the flexible biosensor platform was demonstrated with cyclic bending tests. The sensor performed within 12% CV after 100 bending cycles demonstrating the robustness of the nanostructured ZnO electrochemical sensor platform.

Development of biosensing devices for point-of-care (POC) testing of physiological fluids remain in the high demand for effective diagnostic and prognostic management of diseases. Ever since the introduction of gluocometers and pregnancy test kits, the focus of diagnostic medicine has shifted from centralized hospital clinic-based to home-based testing and has increased awareness about lifestyle amongst people. Over the past decades, glucose monitoring devices have made the testing of physiological fluids by untrained consumers both possible and successful, thus revolutionizing the field of diabetic disease management. Today, major research is geared towards development of similar POC devices for multiple fields including chronic disease diagnosis and prognostic monitoring, food testing, environmental monitoring and many more^1^,^2^,^3^. There is high demand for development of rapid, reliable, easy to use and low cost POC devices with the potential for home-based testing to meet the unmet challenges in disease diagnosis, particularly in cardiovascular disease (CVD) management^4^.

Coronary heart disease (CHD) resulting from atherosclerosis of the heart arteries leads to complications such as Acute Myocardial Infarction or AMI, angina pectoris, and heart failure. About 1 in every 3 deaths from CHD are due to AMI alone^5^. The onset of symptoms in myocardial infarction is usually gradual, over several minutes, and rarely instantaneous^6^. Cardiac troponins are tissue-specific expression markers found in the myocardium because of necrosis and prolonged ischemia to heart muscles. Expression of the two main isoforms of cardiac troponin (cTnT and cTnI) in blood circulation represents the onset of AMI^7^. Therefore, diagnostic tests for cTnT and cTnI are the preferred strategy for risk stratification in patients with non-ST-segment elevation myocardial infarction^8^. Being able to quickly detect these highly specific biomarkers at ultralow concentrations from low patient sample volumes is important as every second counts in making appropriate decision affecting the long-term mortality of the patient. The half-life of cTnT in blood circulation is estimated to be ~2 hours, with longer detection window being due to slower degradation of myofibrillar pool^9^. cTnT can be found in blood within 4 hours following the onset of AMI for up to 3.5 days^10^. Currently there is no POC device available that is designed to specifically monitor cTnT or cTnI levels using peripheral blood samples on a hand-held device similar to a glucometer. Development of a similar monitor for cTnT and cTnI will enable affordable patient testing and monitoring at home or ambulatory environments. Roche’s chemiluminescence based POC system is reported to provide diagnostics results in 12–22 minutes from a sampling of 150 μL of anticoagulated venous or arterial whole blood drawn from the patient [ http://www.cobas.com/home/product/point-of-care-testing/cobas-h-232.html]. Another POC device for cTnI detection in finger pricked blood based on optomagnetic assay by Philips reports a limit of quantification at 20% and 10% CV to be 35 ng/L and 112 ng/L respectively [ http://www.philips.co.uk/healthcare/product/HCNOCTN496/minicare-i20-enabling-near-patient-blood-testing-in-the-acute-care-setting]^11^,^12^. The gap in these hand-held POC devices for cardiac troponin biomarkers that needs to be overcome is the ability to detect ultralow concentrations i.e. ≤1 ng/ml with high accuracy and in very low i.e. ≤20 μL sample volumes of blood/serum.

Detection of biomolecule binding signal can be based on electrochemical, optical i.e. chemiluminescence or reflectance, or magnetic transduction^13^,^14^,^15^. Electrochemical detection methods rely on either voltage or current to detect biomolecular binding and are suitable for implementation in miniaturized electrical biosensor devices^16^. However, in all of these modes of detection, the signals due to biomolecular binding events are of low amplitude and hence methods utilizing nanoparticles^11^ or nanostructured sensing surfaces are utilized for signal amplification to achieve ultrasensitive detection^17^,^18^,^19^,^20^. Among the later, metal oxide nanostructures are attractive candidates for fabricating miniaturized affinity based biosensors with high sensitivity and selectivity. The nanoscale dimensions offer size matching with target biomolecules, increased surface area to volume ratio, and structural morphology providing selective functionalization sites for biomolecular binding. We have previously demonstrated highly specific and ultrasensitive detection of cTnT in patients’ serum samples with LOD at 0.0088 ng/L (i.e. 8.8e-6 ng/mL) on a rigid PCB electrochemical sensor platform integrated with nanoporous membranes^21^,^22^. The four orders higher sensitivity achieved with our electrochemical sensor platform is primarily due to enhancement of biomolecular binding, a direct effect of confinement within the membrane pores on the sensing electrode surface, and resulting in enhanced signal over noise threshold output as characterized using impedance. Non-faradaic electrochemical impedance spectroscopy (EIS)^16^ is a simple and powerful label-free methodology that has been utilized in our previous work and in this paper to measure modulation of charge species resulting from biomolecular binding at the electrode surface towards ultrasensitive detection of biomarkers.

Additionally, in this paper we have investigated the use of porous polyimide substrates to design a flexible disposable strip based biosensor consisting of zinc oxide (ZnO) nanostructures as the sensing electrodes for ultrasensitive and low volume cTnT detection. The facile growth of ZnO nanorods on a variety of substrates combined with its remarkable multifunctional characteristics opens up an entirely new and exciting research direction in the field of electrochemical biosensing^23^,^24^,^25^. Nanostructures of ZnO especially nanowires/nanorods have been extensively investigated for enzymatic biosensing (glucose^25^,^26^, cholesterol^27^, uric acid^28^, etc.) due to the high isoelectric point (IEP = 9.5) of ZnO that favors electrostatic attraction of lower IEP enzymes^23^. Such enzymatic biosensors are focused on monitoring the catalytic activity of the enzymes and such responses have been demonstrated by Zhu *et al*. using ZnO nanoparticles to study the catalytic activity of microperoxidase^29^. These sensors rely on measuring the biochemical metabolites and not all biomolecules (proteins and enzymes) exhibit electro-catalytic activity. However, ZnO as an electrochemical sensing material for affinity based biosensing and low sample volume has scarcely been reported. Hence affinity based biosensing based on antibody-antigen binding interactions is a preferred strategy in development of cardiac biosensors in this work. Investigating the flexible electrochemical sensor fabrication for achieving high sensitivity is deemed necessary for affinity based sensing mechanism. In this paper, we demonstrate ultrasensitive detection is capable at low sample volumes with this flexible ZnO nanostructure based electrochemical sensor platform. The flexible porous polyimide strips allow for very low volumes of fluid absorption within the pores, which in turn ensures more effective conjugation and thus improved sensitivity in the detection of target analyte present in the sample volume on to functionalized ZnO nanostructures as the sensing material. Integration of highly crystalline nanostructures on to flexible substrates is a critical step in the design of the biosensor. Herein we discuss a facile approach to fabricate ZnO nanostructures on to porous polyimide strips using a liquid based hydrothermal synthesis method that is both scalable and has low cost of manufacturing. The fabrication methods used in this work are compatible with roll-to-roll manufacturing guidelines. Therefore, integration of sensing components and fluidic components for sample introduction can all be incorporated within a single flexible strip based POC device.

## Results and Discussion

**Figure 1:**
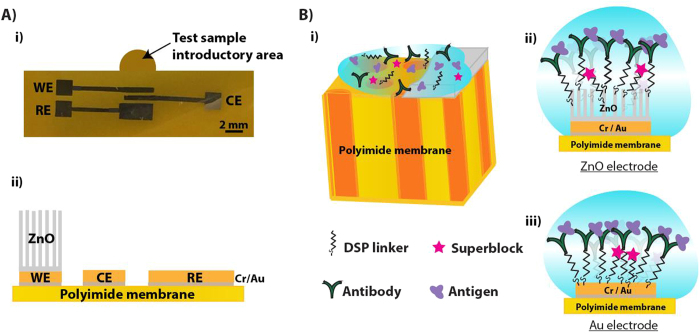
(**A**) Schematic representation of disposable flexible strip based electrochemical biosensor platform (i) Optical image and (ii) electrode material stack. (**B**) Schematic illustration of (i) sample absorption onto the sensing electrode and immunoassay formed at the electrode/electrolyte interface in (ii) ZnO electrodes and (iii) Au electrodes.

In this work, we demonstrate the design, development and testing of a flexible ZnO electrochemical sensor platform for selective and sensitive detection of cTnT in both phosphate buffered saline (PBS) and human serum (HS). This polyimide-based electrochemical diagnostic platform provides promising opportunities to design immunoassays towards detection of multiple biomarkers in a point-of-care or point-of-use device configuration. We used cTnT as the target biomarker to investigate the feasibility of our sensor design and architecture towards the development of flexible and disposable POC cardiac biosensors. The results can then be compared to our previous work on rigid PCB substrates using nanoporous membranes. Our sensor design comprises of metallic gold (Au) electrodes fabricated on porous polyimide membrane substrates with ZnO nanostructures selectively grown on the working electrode (WE). We utilize a three-electrode planar electrochemical cell configuration shown in Fig. 1(A) for this biosensor platform and demonstrate the effect and nature that the electrode material selection for the working electrode has on the biosensing performance. The different working electrode configurations tested were – gold as the sensing surface, ZnO nanotextured seed as the sensing surface, and ZnO nanostructure as the sensing surface respectively. cTnT binding interactions (immunoassay is schematically illustrated in Fig. 1(B)) at the electrode/electrolyte interfaces are utilized for quantification and detection of cTnT in both PBS and HS. Finally, the effects of mechanical handing such as bending of the flexible strips on sensing performance for cTnT detection in HS were characterized.

### Sensor Design and Fabrication

**Figure 2:**
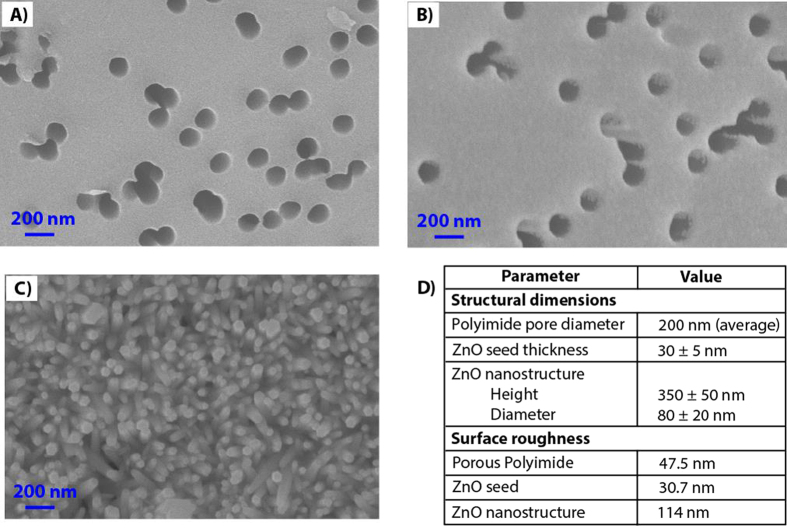
SEM micrograph of (**A**) track-etched porous polyimide membrane substrates, (**B**) ZnO seed deposition on Cr/Au stack, (**C**) ZnO nanostructures grown with 50 mM precursor concentration in an aqueous chemical bath, (**D**) Structural dimensions and surface roughness values of the electrochemical biosensor platform. The pores enable low volume fluid absorption “wicking” thus enhancing biomolecular binding on the functionalized ZnO nanostructures.

ZnO nanostructures offers high surface area, tunable electrical properties and spatial confinement to trap biomolecules and thus can be incorporated into flexible polyimide substrates to design strip based disposable electrochemical biosensors. The SEM micrograph in Fig. 2(A) demonstrates track etched porous architecture of polyimide membranes with about 70% porosity. When ZnO nanotextured seed layer is selectively formed on to metallic electrodes, it also gets deposited inside the pores along its edges as seen in Fig. 2(B). The morphology of grown ZnO nanostructures on this seeded substrate is shown in Fig. 2(C) and measured average height and diameter of the nanostructures are listed as table in Fig. 2(D). It shows SEM images of hexagonal shaped rod-like morphology of ZnO growth. The surface roughness values listed in Fig. 2(D) are obtained from atomic force microscopy (AFM) studies (AFM images are not shown). During the hydrothermal growth, the formation of ZnO nanorods starts with thermal degradation of hexamethylenetetramine (HMTA) additives that helps precipitates hydroxyl ions, which reacts with zinc nitrate to form zinc hydroxyl species. These zinc hydroxyl species nucleate on the seed layers resulting in crystalline growth of highly c-axis oriented ZnO nanostructures. Thus, the morphology of ZnO nanostructures on lattice-mismatched substrates such as porous polyimide membrane is influenced by the seed deposition. In absence of seed layer, random growth of nanostructures with different crystalline orientations and lattice parameters is expected^30^. Figure 2(C) shows that presence of ZnO seed layer is important for c-axis directed and high-density growth of ZnO nanostructures. The other critical parameters that influence the ZnO nanostructure growth in the aqueous chemical bath are the precursor concentration that modulates the density of nanostructures and the reaction time and temperature that influences the growth rate and aspect ratio of nanostructures^31^,^32^. In this work, the ZnO nanostructures synthesized using 50 mM precursor concentration at 80 °C demonstrated an average aspect ratio of ~4 (height: diameter).

The wetting and diffusion characteristics of fabricated sensor strips were studied using physiological buffer, PBS as function of time. For this study, untreated porous polyimide membrane substrates were cut into dimensions matching the form factor of electrochemical biosensor platform using CO_2_ laser. The source of fluid sample addition is shown in Fig. 1(A). The fluid source was chosen to be close to the working electrode to minimize any loss of target biomolecules during diffusion from source to the sensing area surface. The total time required to wet all of the sensing area was observed to be 30 seconds for sample volume of 20 μL. This sample volume was maintained for all the measurements presented in this paper. The porous structure in polyimide substrate allows for the wicking of physiological fluids in contrast to the non-porous polyimide substrates that exhibit minimal fluid interaction properties and this effect was validated with contact angle measurements. The contact angle of porous polyimide membrane and non-porous film was measured to be 22.3° and 75° respectively.

### Electrochemical Impedance Biosensing Characteristics

**Figure 3:**
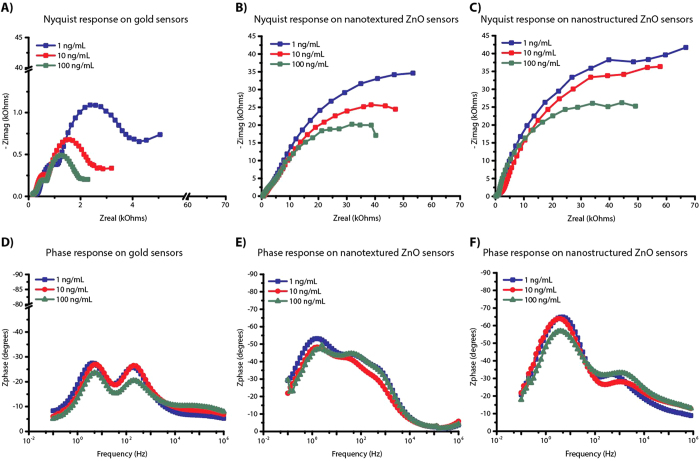
EIS response observed on disposable flexible strip based electrochemical biosensor platform under gold, nanotextured ZnO and nanostructured ZnO electrode configurations for varying concentrations of cTnT biomarker. Comparison of Nyquist and Bode plot for (**A,D**) gold; (**B,E**) nanotextured ZnO; (**C,F**) nanostructured ZnO sensors respectively.

EIS measurements were done with cTnT biomarker diluted in PBS following incubation with increasing cTnT concentrations as a method to characterize the biosensing performance of the functionalized electrochemical sensor platform. Figure 3 compares the measured impedance spectra as Nyquist and Bode plots with the three different working electrode configurations – gold as the sensing surface, ZnO seed as the sensing surface, and ZnO nanostructure as the sensing surface respectively. ZnO seed and hydrothermally grown ZnO nanostructures are hereafter referred to as nanotextured ZnO and nanostructured ZnO respectively. A total of n = 3 replicates were performed for each electrode configuration. Nyquist plots between Z_real_ and Z_imag_ components of impedance indicate the effect of different cTnT concentrations on the charge perturbations at the electrode surfaces and corresponding Bode plot between phase (Z_phz_) and frequency reveal frequency dependence of these electrical components. The information in these plots can therefore be used to understand and quantitate the cTnT binding interactions with concentration.

When a functionalized charged electrode is exposed to an ionic buffer containing biomolecules, the ions will be directly adsorbed at the surface of the electrode forming an electrical double layer (EDL) that can be measured as double layer capacitance (C_dl_) using EIS. The components in the electrolyte extending outside the EDL constitute the solution resistance (R_s_) and the charged interactions at the electrode surfaces constitute the charge transfer resistance (R_ct_). The target biomolecules i.e. cTnT will adhere to the electrode surface through specific binding as shown in Fig. 1B and modulate either or both the C_dl_ and R_ct_ values. Thus, the quantitative measurement of resistive and capacitive components in EIS provides in depth information about the physicochemical changes that occur due to target biomolecular interactions at the electrode surface. The Nyquist plot for gold sensors indicate a decrease in both Z_real_ and Z_imag_ impedances with increasing cTnT concentrations indicating both the resistive and capacitive components are being affected. In contrast, the Nyquist plot for both nanotextured ZnO sensors and nanostructured ZnO sensors indicate mainly a decrease in Z_imag_ impedances with increase cTnT concentrations, indicating only capacitive components are affected and the magnitude of change is higher at lower frequencies. The Bode phase plot for these ZnO sensors establishes the capacitive behavior due to binding events is observed at low frequencies and that due the porous substrate at high frequencies.

**Figure 4:**
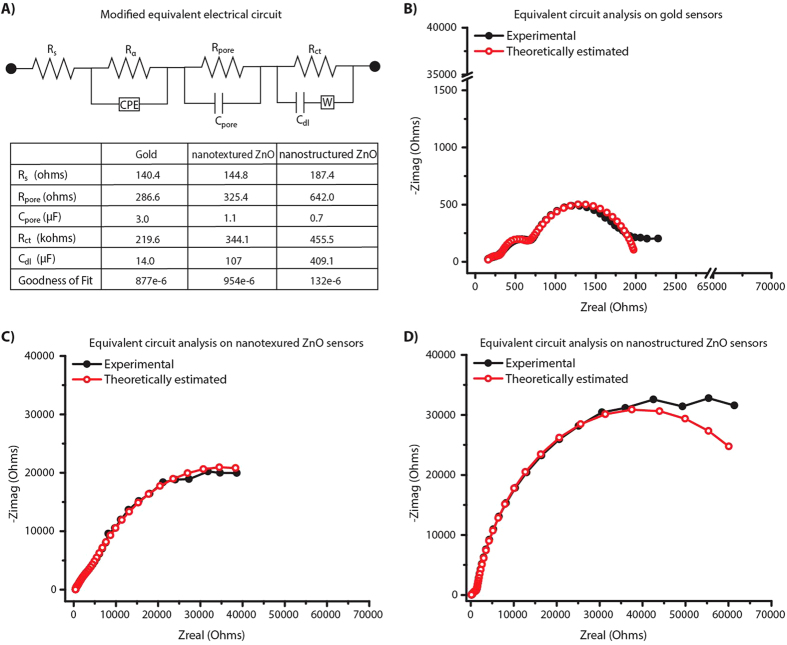
(**A**) Equivalent electric circuit model of the impedance response due to binding events. Table lists electrical parameters obtained using the modified Randles equivalent circuit with multiple iteration solver. Theoretically estimated and experimental Nyquist overlay for (**B**) gold; (**C**) nanotextured ZnO and (**D**) nanostructured ZnO electrode configurations at 100 ng/mL cTnT antigen concentration.

The complex plane impedance plots in Fig. 3 indicates the presence of more than one relaxation time constants. We fitted the obtained EIS plots with the modified Randles equivalent circuit^16^,^33^,^34^ model shown in Fig. 4(A). Using Gamry Echem analyst, the experimentally obtained impedance spectra for 100 ng/mL was fitted with the equivalent circuit model as shown in Fig. 4(B–D). In electrode configuration with gold metallic electrodes as in Fig. 4(B), the first semicircle indicates the polarization impedance of electrolyte within the porous structures of the polyimide substrate at high frequencies. This is represented by a combination of dominant pore resistance (R_pore_) and capacitance (C_pore_) in parallel in circuit model. The second semicircle at low frequencies is indicative of changes within the EDL and hence is represented as a parallel combination of R_ct_ and C_dl_. Analysis of the Bode plot confirms the frequency dependence of circuit combination of R_ct_ and C_dl_. The resistive element at very high frequencies is due to R_s_. However, in the presence of ZnO the overall impedance is higher than the gold electrode configuration. In the nanotextured ZnO electrode configuration as shown in Fig. 4(C), the impedance due to porous behavior is dominated by the inherent electrical properties in ZnO and is difficult to separate the contributions due to cTnT antigen binding and ZnO. Whereas in presence of nanostructured ZnO electrode (shown in Fig. 4(D)), the dominant polarization impedance due to cTnT interactions is evident at the low frequencies. Table in Fig. 4(A) shows that with the formation of ZnO nanostructures, the contribution from C_pore_ is reduced from 3.0 μF to 0.7 μF. The C_dl_ measured in presence of nanostructured ZnO surface is about 4x and 3x times higher than the planar gold and nanotextured ZnO respectively. In addition to these electrical components, a resistive component (R_α_) and a constant phase element (CPE) represents the inhomogeneity in the electrode surface^35^ and does not change with different electrode configurations. Though signal amplification is achieved with both nanotextured ZnO and nanostructured ZnO electrode surfaces compared to the gold electrode surface, the nanotextured electrode surface exhibits more of microelectrode behavior while the nanostructured ZnO surface exhibit a true nanoelectrode behavior and therefore is better suited for specific detection of cTnT antigen. From the plots in Figs 3 and 4, it is clear that the frequency range of operation for understanding the capacitive dominant electrical components representing the biosensor performance is between 1 Hz and 50 Hz.

### Sensor Analytical Performance

**Figure 5:**
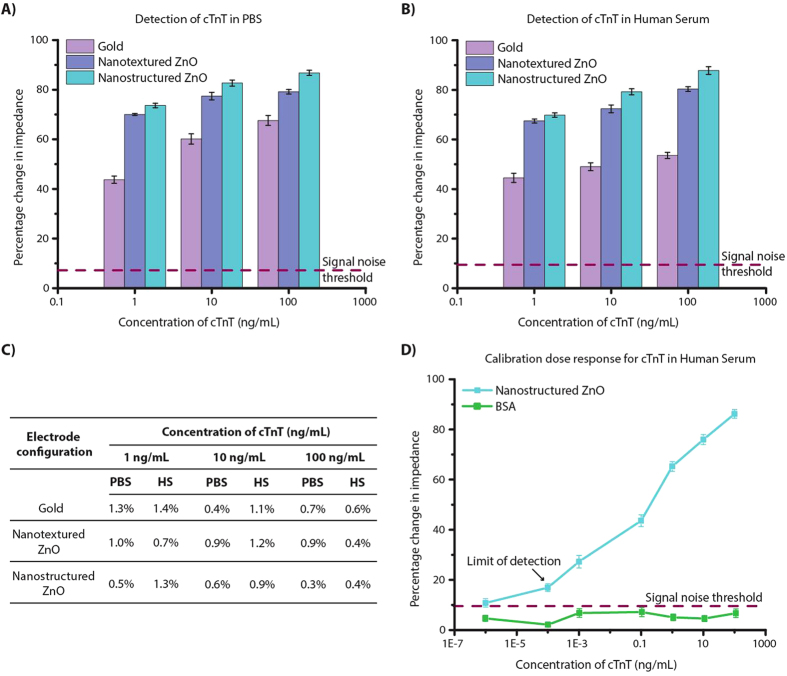
Calibration dose response curve for cTnT antigen concentrations spiked in (**A**) PBS and (**B**) HS for varying electrode configurations and (**C**) %CV observed at each cTnT antigen concentration. (**D**) Detailed dose response curve for cTnT antigen concentrations on nanostructured ZnO electrode configuration. BSA was chosen for cross-reactivity and selectivity validation. Error bars represent 2SD from n = 3 replicates.

A calibration dose response curve is built between cTnT antigen concentration and percentage change in impedance calculated from the EIS measurements. We performed dose response study for 1, 10 and 100 ng/mL cTnT antigen concentrations spiked in both PBS and HS buffer on α-cTnT immobilized sensing surfaces. The sensor baseline measurement was taken with zero concentration of cTnT antigen i.e. test buffer on antibody immobilized sensor device. The PBS or HS step post blocking of unoccupied DSP sites correspond to zero dose baseline measurement. The change in impedance with increasing cTnT antigen concentrations is calculated from this baseline impedance. The frequency at which the capacitive binding events dominated with maximum impedance change was observed at 2.5 Hz. The percentage change in impedance was chosen to represent the calibration dose response observed for discussed electrode configurations. Fig. 5(A,B) shows the calibration curves for cTnT antigen spiked in PBS and HS respectively for the three different electrode configurations. We observed decrease in impedance with increasing concentration of cTnT antigen with all three-electrode configurations. The dynamic range of impedance signal obtained for cTnT antigen diluted in PBS and HS was 68% and 53% on gold, 79% and 80% on nanotextured ZnO and 85% and 86% on nanostructured ZnO surface respectively. The % CV for each cTnT antigen concentrations was calculated and is shown in Fig. 5(C). To quantify the noise in signal due to the buffer, the sensors were washed thrice with the test buffer between each assay step and impedance measurements were taken. The change in impedance between pre and post buffer wash in superblock treated sensor is estimated as signal noise for the developed sensor platform. The signal noise threshold is estimated to be 3x times the signal noise for any electrical biosensor and it was 7.19% with PBS and 9.52% with HS. The calibration dose response curve was extended for the nanostructured ZnO platform as shown in Fig. 5(D) to understand the sensor performance capability. The limit of detection (LOD) in HS was identified at 0.0001 ng/mL or 0.1 ng/L with 7% CV which is three orders lower than that reported on current handheld POC devices for cardiac troponin detection^12^ [ http://www.cobas.com/home/product/point-of-care-testing/cobas-h-232.html, http://www.philips.co.uk/healthcare/product/HCNOCTN496/minicare-i20-enabling-near-patient-blood-testing-in-the-acute-care-setting]. The selectivity of anti-cTnT immobilized biosensor platform was tested with bovine serum albumin (BSA), a most abundant protein found in human blood plasma and which may cross-react with the amine groups in the antibody side chains. The maximum percentage change in impedance observed with BSA as shown in Fig. 5(C) was less than 10% that is much lower than the change observed with lowest cTnT antigen concentration and the signal noise threshold.

### Effect of Strain on Sensor Performance

**Figure 6:**
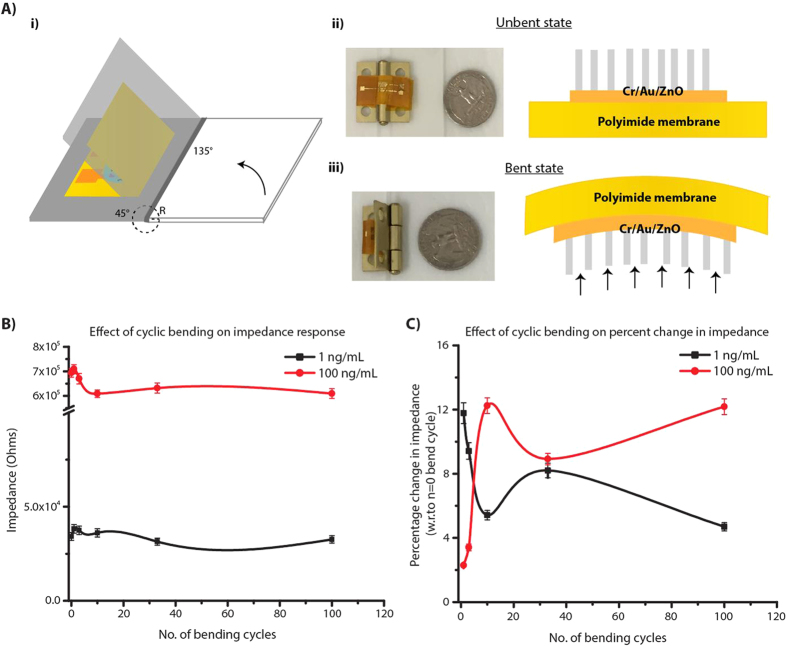
(**A**) Schematic of the cyclic bending test on ZnO nanostructured sensor stack and optical image of the home-built apparatus used, (**B**) absolute impedance (Zmod) vs. no. of bending cycles and (**C**) percentage change in impedance with respect to n = 0 bending cycle vs. no. of bending cycles.

A mechanistic insight into the operation of electrochemical sensors under strain could provide new pathways to eliminate or enhance the coupling of strain on the biomolecular binding efficacy, the electronic signal transduction, and the structural integrity of the sensor stack thus enabling robust and reliable, ultrasensitive detection of biomolecules. Among these effects, the structural integrity of the sensor stack and the biosensor response needs to be ensured before delving into the effects of strain on biomolecular interactions occurring within the electrode/electrolyte interfaces. Electrode material on flexible substrates can experience electrical failure due to repeated strain from handling and bending^36^,^37^,^38^. The electrical behavior of the flexible ZnO nanostructured devices was characterized during the systematic application of bending strain. When a flexible substrate material is bent, it experiences tensile stress on the outer surface and the compressive stress on inner surface with no stress along its neutral plane^35^,^37^,^39^. A schematic illustration of flexible electrochemical platform experiencing strain due to cyclic bending is illustrated in Fig. 6(A). The strain (ɛ) in the device due to cyclic bending was evaluated from the bending radius (R) by quantitative analysis using the Equation (1)^37^,^38^:


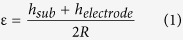


where, h_*sub*_ is the substrate thickness and h_*electrode*_ is the electrode material thickness respectively. The thickness of the electrode material is about three orders of magnitude smaller compared to thickness of porous polyimide substrate and therefore does not play a role in the magnitude of the strain. Cyclic bending experiments were performed by fixing the superblock treated flexible sensor surface to home-built bending apparatus and then subjecting them to bend inwards at an angle of 45°. An optical image of bending apparatus with the flexible electrochemical sensor is shown in Fig. 6(A). The bending radii (R) on the sensor with our apparatus was 9 mm resulting in a bending strain of 0.2% applied at each cycle. Thus, for each bending cycle the device was systematically taken from 0% strain (unbent state) to 0.2% (bent state) and back to 0% (unbent state). The impedance measurements were taken post incubation of cTnT antigen and prior to the start of cyclic bending which corresponds to n = 0 bending cycle. The sensors were subjected to cyclic bending i.e. n = 1, 3, 10, 33, and 100 bending cycles and impedance measurements were taken at the end of each of these cycles with sensors at 0% strain position (unbent state). These measurements were respectively carried out for each cTnT antigen doses on separate sensor platforms and up to a total of n = 100 bending cycles. Figure 6(B) is plots of absolute impedance (Z_mod_) vs. no. of bending cycles and percentage change in impedance with respect to n = 0 bending cycle vs. no. of bending cycles. Z_mod_ plot shows the impedance regimes are different for the two dose concentrations of 1 ng/mL and 100 ng/mL and after n = 10 cycles changes very little. The percentage change in impedance plot shows an initial inflexion for up to n = 10 cycles followed by a trend towards stabilization for both the dose concentrations. We interpret this as stabilization of the strain in the multi-layer sensor stack (i.e. ZnO nanostructure on Cr/Au electrode stack) and that the changes in impedance output are within the acceptable % CV desired for biosensor performance.

### Conclusions

In this manuscript, the role of porous flexible polyimide substrate on electrochemical biosensing performance was evaluated. The pores in the flexible substrate allows for effective wicking of low volumes (≤20 μL) of fluid and confinement of target biomolecular species in close proximity to the sensing electrode surfaces. Such confinement mechanisms are especially relevant for biosensors that employ affinity based mechanisms for detection of target species. While this work focused on EIS as the method of detection, the confinement based enhancement of signal is expected to also benefit other modes of detection. By using different electrode materials, we demonstrated metal oxide semiconducting performs better than metallic thin films for signal amplification of biomolecular interactions and that nanostructured metal oxide semiconducting films can significantly enhance the sensitivity of biomolecular detection. A three order lower limit of detection compared to current handheld POC diagnostic devices was demonstrated for cardiac troponin biomarker, cTnT in HS. Thus, flexible porous substrates with functionalized semiconducting surfaces allows for enhanced charge transfer on the sensing surfaces and is therefore suited for rapid and sensitive, low-cost strip based biosensors in POC diagnostic devices. Studies were conducted to understand the influence of nanostructure growth on porous substrate towards understanding ease of manufacturability. Finally, the effects of mechanical handing such as cyclic bending of the flexible strips was studied to demonstrate minimal loss of sensing performance. Further mechanistic studies are needed to understand the effect of strain on biomolecular binding efficacy for establishing robustness and reliability of such flexible electrochemical sensor platforms.

## Experimental Section

### Preparation of Sensing Electrodes

A schematic representation of sensor design is shown in Fig. 1(A). A three-electrode sensor with 25 nm Cr/ 125 nm Au was e-beam deposited on 50 mm diameter, 25 μm thick porous polyimide membrane through a patterned stencil mask. Subsequent to the deposition, the patterned membranes were subjected to secondary stencil mask that allows selective deposition of ZnO only at the working electrode (WE). ZnO seed layer was deposited in conventional RF-magnetron sputter using ZnO target of 99.99% purity under 12 sccm Ar plasma with no oxygen and power at 50 W. The deposition was carried at base pressure of 15 mTorr for about 30 minutes. The thickness of deposited ZnO seed layer was validated using Veeco Dektak 8 profilometer and was found to be 30 ± 5 nm.

### Synthesis of ZnO nanostructures

The synthesis of ZnO nanorods at seed deposited WE was carried out through hydrothermal method. This liquid phase method consists of equimolar concentration (50 mM) of zinc nitrate hexahydrate and hexamethylene tetramine (HMTA) as precursors in aqueous chemical bath at temperatures lesser than boiling point of water. The nanorods were grown on seed layer via nucleation process at 80 °C for 30 minutes under continuous stirring at 300 rpm. Following the synthesis, the samples were rinsed with deionized water and dried in air for further surface and electrochemical characterization studies. Thus grown nanorods were about 350 ± 50 nm in length and 80 ± 20 nm in diameter.

### Immobilization of Proteins

The immobilization of capture antibody on the sensing surface (i.e. gold, nanotextured ZnO and nanostructured ZnO) was achieved by covalent attachment of crosslinking molecule. The goal here was to keep the biomolecules bound to the sensing area where biochemical interactions are converted in to a detectable electrical output signal response. A 10 mM dithiobis (succinimidyl) propionate (DSP; Pierce Biotechnologies, IL) dissolved in dimethyl sulfoxide (DMSO; Fisher Scientific, TX) was allowed to interact with ZnO nanorods for 2 hours followed by treatment with 1 μg/mL anti-cTnT antibody (US Biological, MA) in PBS for 15 minutes. The cleavable disulphide bridge in DSP’s 12 spacer arm interacts with Au/ ZnO and NHS ester group at its terminal end interacts with primary amine residues in antibody forming a strong thiol and amide linkage respectively. The unbound sites in DSP was then blocked by treatment with Superblock (Fisher Scientific, TX) to eliminate non-specific interactions with target antigen. Doses 1, 10 and 100 ng/mL of cTnT antigen in PBS/HS were tested on thus prepared sensing surface starting with lowest concentration and impedance measurements were taken after 15 minutes of incubation. The cTnT doses tested on nanostructured ZnO to build calibration curve was 1E-6, 1E-4, 1E-3, 0.1, 1, 10 and 100 ng/mL. Detection volume and fluid release into sensing area was identified through basic fluid wick test with colored PBS buffer. The schematic representation of immunoassay protocol is shown in Fig. 1(B). A total of n = 3 replicates were performed and the turnaround time for cTnT detection was less than 20 minutes.

### Electrochemical Measurements

EIS was used for evaluating the surface charge perturbations due to biomolecular binding interactions at the sensing area. All electrochemical measurements were performed using a Gamry Reference 600 potentiostat (Gamry Instruments, PA) at room temperature over the frequency range 0.1 Hz to 1 MHz. A typical three-electrode setup, consisting of Au/ZnO as working electrode (WE), Au as both counter electrode (CE) and reference electrode (RE) was used. The surface area of WE:CE:RE were designed in ratio of 1:1:4 to ensure the output signal response due to binding events at WE. The dimensions of connecting pads were chosen such that it allows impedance matching with the alligator clip connectors. The sensors were fixed on glass substrates using adhesive tapes at the corners for support and to prevent curling and thus the deformation of the device. The stability of the electrode design for biosensing was evaluated using open and short circuit potential measurements and the corresponding data is shown as Figure S1 in the supporting information. To understand the electrochemical performance of the developed sensor platform when subjected to mechanical bending, measurements were taken after being bent to approximately 9 mm radius of curvature. The sensors were fixed on home-built bending apparatus and the cyclic bending were performed up to 100 cycles.

## Additional Information

**How to cite this article**: Shanmugam, N. R. *et al*. Ultrasensitive and low-volume point-of-care diagnostics on flexible strips – a study with cardiac troponin biomarkers. *Sci. Rep.* **6**, 33423; doi: 10.1038/srep33423 (2016).

## Supplementary Material

Supplementary Information
