# Supplementary material for: Ultrasensitive and low-volume point-of-care diagnostics on flexible strips – a study with cardiac troponin biomarkers
Source: Sci Rep. 2016 Sep 16;6:33423. doi: 10.1038/srep33423 (PMC5025736; doi:10.1038/srep33423)
Supplement: Supplementary Information [file srep33423-s1.docx]

**Ultrasensitive and low-volume point-of-care diagnostics on flexible strips – a study with cardiac troponin biomarkers**

Nandhinee Radha Shanmugam^1^, Sriram Muthukumar^2^ and Shalini Prasad^1, †^

^1^Department of Bioengineering, University of Texas at Dallas, Richardson, TX 75080

^2^EnLiSense LLC, 1813 Audubon Pond Way, Allen, TX 75013

^†^Corresponding author, Email: [shalini.prasad@utdallas.edu](mailto:shalini.prasad@utdallas.edu)

***Supporting Information***

**Electrode stability measurements**

The performance of the sensor electrodes were evaluated under open circuit and short circuit conditions and measurements are shown as graph below. The potential of the electrodes measured in absence of any electrical connection is the open circuit potential and in presence of electrical connection as short circuit potential. The electrical connection between the electrodes were established using 0.15 M PBS at pH 7.0. It was found that with addition of PBS, the potential shifted more towards negative side mainly due to pH of the added solution buffer. The potential increases with time before it reaches its steady state (~300 seconds). Based on these potential measurements we can infer that the sensor dimensions enable performing the sensing assay as described in the paper under stable conditions.


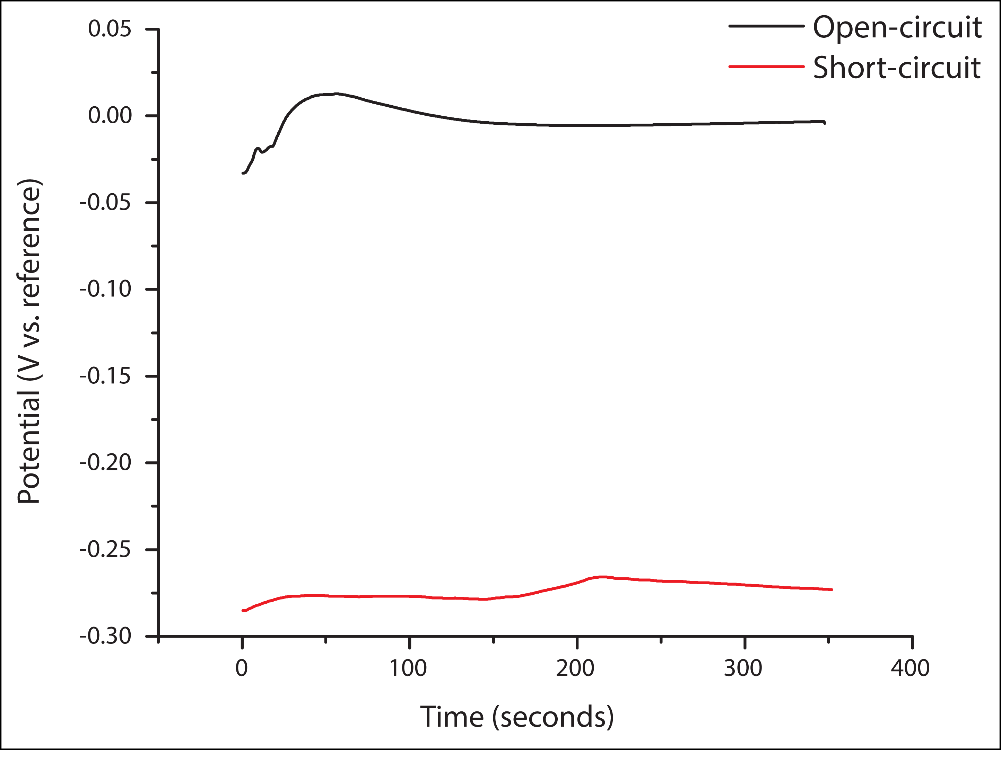


Figure S1. Open and short circuit potential measurements of the disposable polyimide based electrochemical biosensor.
